# The International/Canadian Hereditary Angioedema Guideline

**DOI:** 10.1186/s13223-019-0376-8

**Published:** 2019-11-25

**Authors:** Stephen Betschel, Jacquie Badiou, Karen Binkley, Rozita Borici-Mazi, Jacques Hébert, Amin Kanani, Paul Keith, Gina Lacuesta, Susan Waserman, Bill Yang, Emel Aygören-Pürsün, Jonathan Bernstein, Konrad Bork, Teresa Caballero, Marco Cicardi, Timothy Craig, Henriette Farkas, Anete Grumach, Connie Katelaris, Hilary Longhurst, Marc Riedl, Bruce Zuraw, Magdelena Berger, Jean-Nicolas Boursiquot, Henrik Boysen, Anthony Castaldo, Hugo Chapdelaine, Lori Connors, Lisa Fu, Dawn Goodyear, Alison Haynes, Palinder Kamra, Harold Kim, Kelly Lang-Robertson, Eric Leith, Christine McCusker, Bill Moote, Andrew O’Keefe, Ibraheem Othman, Man-Chiu Poon, Bruce Ritchie, Charles St-Pierre, Donald Stark, Ellie Tsai

**Affiliations:** 1 University of Toronto, Toronto, ON Canada; 2 HAE Canada, Notre Dame des Lourdes, MB Canada; 3 Department of Medicine, Queen’s University, Kingston, ON Canada; 4 Department of Medicine, Laval University, Quebec City, QC Canada; 5 Division of Allergy and Clinical Immunology, St. Paul’s Hospital, Department of Medicine, University of British Columbia, Vancouver, BC Canada; 6 Department of Medicine, McMaster University, Hamilton, ON Canada; 7 Department of Medicine, Dalhousie University, Halifax, NS Canada; 8 University of Ottawa Medical School, Ottawa, ON Canada; 9 Goethe-Universität Frankfurt am Main, Frankfurt am Main, Germany; 10 Department of Internal Medicine, University of Cincinnati, Cincinnati, OH USA; 11 Department of Dermatology, University Hospital of the Johannes Gutenberg-University of Mainz, Mainz, Germany; 12 Hospital La Paz Institute for Health Research, Madrid, Spain; 13 Department of Internal Medicine, Universita degli Studi di Milano, Ospedale L. Sacco, Milan, Italy; 14 Departments of Medicine and Pediatrics, Penn State University, Hershey, PA USA; 15 3rd Department of Internal Medicine, Faculty of Medicine, Semmelweis University, Budapest, Hungary; 16 Laboratory of Clinical Immunology, Faculdade de Medicine ABC, Sao Paulo, Brazil; 17 Campbelltown Hospital, Western Sydney University, New South Wales, Australia; 18 Addenbrooke’s Hospital, Cambridge and University College Hospital, London, England UK; 19 University of California, San Diego, San Diego, CA USA; 20 Moncton Hospital, Moncton, NB Canada; 21 Division of Allergy and Clinical Immunology, Centre hospitalier universitaire de Québec, Laval University, Quebec City, QC Canada; 22 HAE International (HAEi), Horsens, Denmark; 23 HAE International (HAEi), Fairfax, VA USA; 24 Institut de recherches cliniques de Montréal, Montreal, QC Canada; 25 Toronto Allergy Group, Toronto, ON Canada; 26 Southern Alberta Rare Blood and Bleeding Disorders Program, Foothills Medical Centre, University of Calgary, Calgary, AB Canada; 27 Division of Pediatrics, Faculty of Medicine, Memorial University, St John’s, NF Canada; 28 Janeway Children’s Health and Rehabilitation Centre, Memorial University, St John’s, NF Canada; 29 Division of Clinical Immunology and Allergy, Department of Medicine, Western University, London, ON Canada; 30 Division of Clinical Immunology and Allergy, Department of Medicine, McMaster University, Hamilton, ON Canada; 31 Department of Medicine, University of Toronto, Oakville, ON Canada; 32 Department of Immunology, McGill University Health Centre, Montreal, QC Canada; 33 Department of Medicine, Western University, London, ON Canada; 34 College of Medicine, University of Saskatchewan, Regina, SK Canada; 35 Departments of Medicine, Pediatrics and Oncology, University of Calgary Cumming School of Medicine, Calgary, AB Canada; 36 Departments of Medicine and Medical Oncology, University of Alberta, Edmonton, AB Canada; 37 L’angio-oedème héréditaire du Québec, Quebec City, QC Canada; 38 Department of Medicine, University of British Columbia, Vancouver, BC Canada; 39 Department of Internal Medicine, Queen’s University, Kingston, ON Canada

**Keywords:** Hereditary angioedema, Guideline, Recommendations, Pediatrics, Pregnancy, Acute attacks, Short-term prophylaxis, Long-term prophylaxis, Quality of life, Patient registry

## Abstract

This is an update to the 2014 Canadian Hereditary Angioedema Guideline with an expanded scope to include the management of hereditary angioedema (HAE) patients worldwide. It is a collaboration of Canadian and international HAE experts and patient groups led by the Canadian Hereditary Angioedema Network. The objective of this guideline is to provide evidence-based recommendations, using the GRADE system, for the management of patients with HAE. This includes the treatment of attacks, short-term prophylaxis, long-term prophylaxis, and recommendations for self-administration, individualized therapy, quality of life, and comprehensive care. New to the 2019 version of this guideline are sections covering the diagnosis and recommended therapies for acute treatment in HAE patients with normal C1-INH, as well as sections on pregnant and paediatric patients, patient associations and an HAE registry. Hereditary angioedema results in random and often unpredictable attacks of painful swelling typically affecting the extremities, bowel mucosa, genitals, face and upper airway. Attacks are associated with significant functional impairment, decreased health-related quality of life, and mortality in the case of laryngeal attacks. Caring for patients with HAE can be challenging due to the complexity of this disease. The care of patients with HAE in Canada, as in many countries, continues to be neither optimal nor uniform. It lags behind some other countries where there are more organized models for HAE management, and greater availability of additional licensed therapeutic options. It is anticipated that providing this guideline to caregivers, policy makers, patients, and advocates will not only optimize the management of HAE, but also promote the importance of individualized care. The primary target users of this guideline are healthcare providers who are managing patients with HAE. Other healthcare providers who may use this guideline are emergency and intensive care physicians, primary care physicians, gastroenterologists, dentists, otolaryngologists, paediatricians, and gynaecologists who will encounter patients with HAE and need to be aware of this condition. Hospital administrators, insurers and policy makers may also find this guideline helpful.

## Background

Hereditary angioedema (HAE) results in random and often unpredictable attacks of painful swelling typically affecting the extremities, bowel mucosa, genitals, face and upper airway [1]. Attacks are associated with significant functional impairment, decreased health-related quality of life (HRQoL), and mortality in the case of laryngeal attacks [2, 3].

HAE can be categorized into 3 different types including HAE with deficit C1-inhibitor levels (HAE-1), HAE with dysfunctional C1-inhibitor (HAE-2), and HAE with normal C1-inhibitor function (HAE nC1-INH) previously referred to as type 3 (Table 1). HAE-1 and HAE-2 are autosomal dominant conditions with a combined estimated prevalence of approximately 1:50,000, although 25% of patients may have no family history [4, 5]. HAE-1 is the most prevalent, representing approximately 85% of cases, and results from low antigenic and functional levels of C1-INH. HAE-2 accounts for approximately 15% of cases and is associated with a normal C1-INH protein concentration but impaired C1-INH function [6, 7]. C4 is reduced in 98% of cases for both HAE-1 and HAE-2, and nearly 100% of the time during an attack [6]. The swelling in HAE-1/2 is a result of impaired regulation of bradykinin synthesis [8]. Bradykinin is a nonapeptide kinin formed from high molecular weight kininogen by the action of plasma kallikrein. Bradykinin is a very powerful vasodilator that increases capillary permeability, constricts smooth muscle, and stimulates pain receptors [4, 5].

**Table 1:** Laboratory findings in hereditary angioedema [9–11]

Function	C4	C1-INH antigen	C1-INH
HAE-1	↓	↓	↓
HAE-2	↓	normal or ↑	↓
HAE-nC1INH variants coagulation factor XII angiopoietin-1 plasminogen unknown	normal	normal	normal

HAE nC1-INH is much less prevalent than HAE-1 and HAE-2, and the true prevalence is not known. Identifying patients with HAE nC1-INH is more difficult than identifying those with HAE-1/2 due to the lack of accessible and available assays, including genetic testing for diagnosis. While HAE nC1-INH presents similarly, its pathogenesis has not been clearly defined. Its causes can be subdivided into four groups: HAE-FXII, HAE-ANGPT1, HAE-PLG, and HAE-UNK. Four distinct variants in the gene coding for coagulation factor XII (FXII) can lead to HAE-FXII. One of these variants, Thr328Lys, is far more common. These variants create a cleavage site for plasmin, which facilitates the activation of FXII and the generation of bradykinin. Several aspects of the pathogenesis and the penetrance of HAE-FXII remain unclear including the role of estrogens [12]. In HAE-ANGPT1, a variant in the angiopoietin-1 gene (*ANGPT1*) impairs its ability to limit vascular permeability. In HAE-PLG, the plasminogen gene (*PLG*) is affected, but the mechanism of action is unknown. HAE-FXII accounts for about one third of HAE nC1-INH cases [13–15] while the majority are HAE-UNK. The term HAE-UNK refers to HAE nC1-INH where both the cause and pathogenesis remain unknown [11]. A previous international consensus group published criteria to make the diagnosis of HAE nC1-INH [16]. These included: (1) a history of recurrent angioedema in the absence of concomitant hives or use of medication known to cause angioedema; (2) documented normal or near normal C4, C1-INH antigen, and C1-INH function; and (3) either a genetic variant associated with the disease, or a family history of angioedema and documented lack of efficacy of chronic high-dose antihistamine therapy.

Management of HAE can be divided into various approaches. The aim of acute treatment of HAE attacks, also referred to as “on-demand therapy”, is to minimize their severity and duration, including potentially fatal upper airway edema and associated impairment of quality of life (QoL). Short-term prophylaxis (STP) refers to treatment meant to minimize the risk of attacks when exposure to a potential or known trigger is anticipated. Long-term prophylaxis (LTP) refers to ongoing treatment of HAE aimed at minimizing the overall number, frequency and/or severity of attacks. The details of specific therapies for these treatment approaches will be discussed in the sections that follow. In addition to the evidence behind the proposed recommendations, the “Clinical Considerations” section following each recommendation provides a practical clinical context to assist clinicians in managing individual patients.

### Scope and purpose

The objective of this guideline is to provide evidence-based recommendations for the management of patients in Canada and internationally with HAE-1, HAE-2, and HAE nC1-INH. This includes the treatment of attacks, STP, LTP, and recommendations for self-administration, individualized therapy, QoL and comprehensive care. New to the 2019 international/Canadian version of this guideline are sections covering the diagnosis and recommended therapies for acute treatment in patients with HAE nC1-INH, as well as sections on pregnant and paediatric HAE patients, patient associations and an HAE registry.

The care of patients with HAE in Canada, as in many countries, is neither optimal nor uniform. It lags behind some other countries where there are more organized models for HAE management and greater availability of additional licensed therapeutic options [17, 18]. It is anticipated that providing this international/Canadian guideline to caregivers, policy makers, patients, and advocates will not only optimize the management of HAE, but also promote the importance of individualized care.

### Intended audience

The primary target users of this guideline are healthcare providers who are managing patients with HAE-1, HAE-2, and HAE nC1-INH. Other healthcare providers who may use this guideline are emergency and intensive care physicians, primary care physicians, gastroenterologists, dentists, otolaryngologists, paediatricians, and gynaecologists who will encounter patients with HAE and need to be aware of this condition. Hospital administrators, insurers, and policy makers may also find this guideline helpful.

## Methods

### Committee Members, Guideline Authors and Conference Participants

The Canadian Hereditary Angioedema Guideline Committee (“the Committee”) is a working committee under the umbrella of the Canadian Hereditary Angioedema Network (CHAEN)/Réseau Canadien d’angioédème héréditaire (RCAH) http://chaen-rcah.ca/. Members of this committee included members from CHAEN/RCAH across Canada, as well as the President of the Canadian HAE Patient Organization, Hereditary Angioedema Canada (HAEC). The Committee was responsible for defining the scope and purpose of the guideline and choosing the international participants. International participants were selected based on their relevant expertise in HAE and its management, and guideline priorities including self-administration, individualized therapy, HRQoL, and comprehensive care, in addition to new sections on pregnant and paediatric HAE patients, and HAE nC1-INH. There was also representation from Angio-oedème Héréditaire du Québec (AOHQ) and members of the global HAE patient group, HAE International (HAEi). Identified experts were asked to present a summary of the evidence related to these areas at the CHAEN/RCAH Guideline Conference (“the Conference”).

Guideline Authors represented healthcare providers who are the intended users of this guideline. They included the Committee, international experts, registered members of CHAEN, the President of HAEC and AOHQ Canada and their designates, and the Executive Director and the President of HAEi. Representatives from Héma-Québec and Canadian Blood Services were invited as observers. An invitation was extended to representatives of the provincial/territorial blood coordinating offices, but was declined. All Guideline Authors were asked to submit standard ICMJE conflict of interest forms, which were vetted by an independent reviewer for potential conflicts. “Conference Participants” were the Guideline Authors who were able to attend the Conference. “We” refers to Guideline Authors who voted on the recommendations (see Recommendation Development and Approval below for further details).

### Identifying the evidence

A librarian from the Centre for Effective Practice conducted a systematic search using Ovid MEDLINE on June 27, 2018, based on the predefined scope as described in Appendix 1. The search was designed to identify the current evidence on long- and short-term prophylaxis and acute treatment of attacks in patients of any age diagnosed with HAE-1, HAE-2, or HAE nC1-INH. The search was repeated on November 4, 2018 using the same search strategy to ensure that the most recent evidence was considered at the Conference held from November 30 to December 2, 2018.

Outcomes of interest included frequency or severity of attacks, symptom relief, or quality of life measures as reported or measured by the subject or investigator. Studies were limited to English language publications that were published and indexed in MEDLINE since the search was conducted for the 2014 Canadian Hereditary Angioedema Guideline in October 2013 [19].

198 unique results were identified in the search, and two reviewers independently applied the predetermined inclusion criteria to the titles and abstracts. If either reviewer indicated that a result required further consideration at the title and abstract review stage, the full text document was retrieved and reviewed by both reviewers.

106 results were retrieved and reviewed in full text by both reviewers. Any disagreements between the reviewers were discussed electronically until consensus was reached. Ten relevant randomized controlled trials and 15 lower-quality studies met the inclusion criteria and were included in evidence tables. The complete search strategy and inclusion criteria are provided in Appendix 1.

All 416 results previously identified in the October 2013 search were examined to determine if any met the inclusion criteria for the new populations of interest for this guideline (i.e., pregnant and paediatric populations). One result identified in the 2013 search met the inclusion criteria and was included in the evidence table for lower-quality studies.

To ensure the comprehensiveness of the evidence base, the Committee was invited to suggest additional papers for consideration. One study that was published after the November 2018 search, which met the inclusion criteria, was identified by the Committee and added to the evidence table for randomized controlled trials.

With the addition of these two results, 11 randomized controlled trials and 16 lower-quality studies without randomization or blinding met the inclusion criteria, and were entered into evidence tables.

### Summarizing and evaluating the evidence

Key information from the included studies such as study design, number of patients, outcome measures, side effects and funding source was extracted into evidence tables for each intervention (see Additional files 1, 2). Evidence tables were provided to the Committee and were available for reference at the Conference.

Criteria for determining Levels of Evidence and Strength of Recommendation were adapted from the GRADE system [20–22], and the process was based primarily on the Journal of Clinical Epidemiology’s 2011–2013 series of articles describing the GRADE methodology. The method applied here involved evaluating the quality of each study, and then evaluating the studies together to assign a Level of Evidence based on the collection of studies. Each identified randomized controlled trial was assessed by two reviewers for quality using the Cochrane Risk of Bias Tool [23]. A third reviewer resolved any disagreements. Randomized trials were initially rated as high-quality level of evidence, with quality being downgraded for evidence of bias on the Cochrane tool if there was evidence of inconsistency (Appendix 2: Table 5). Non-randomized, non-blinded trials were considered to be low-quality evidence.

Appendix 2 provides additional detail on how quality was assessed and the criteria used to determine the Strength of Recommendation. The quality ratings were presented at the Conference during the discussion of draft recommendations.

### Recommendation development and approval

The Committee reviewed recommendations that were unlikely to change from the last guideline. The Chair then sent out these recommendations for pre-approval by all voting Guideline Authors prior to the Conference. For all remaining topics, content experts were assigned specific topic areas and were asked to review the provided evidence tables relevant to their topic and present the body of evidence for consideration at the Conference. After the summary was presented, Conference Participants were provided an opportunity to discuss the literature. Following this discussion, the draft recommendation was presented and the group discussed the specific wording of the recommendation before voting anonymously via electronic voting to agree or disagree with the recommendation, or abstain. If 80% consensus was not reached, there was additional group discussion, the recommendation was rephrased, and a new vote conducted. This process was conducted a maximum of three times. If 80% consensus was not reached, it was determined that the group was unable to reach consensus.

Once Conference Participants approved the phrasing of a recommendation, the guideline methodologist presented the proposed Level of Evidence (High, Moderate, Low, Very Low, or Consensus). The Level of Evidence was then discussed, revised if necessary, and similarly voted on as outlined above.

The suggested Strength of Recommendation (Strong or Weak) was then presented to the group. The guideline methodologist proposed a Strength of Recommendation based on the Level of Evidence, the balance between desirable and undesirable effects, values, and preferences. These factors were discussed amongst the group before voting to accept the proposed Strength of Recommendation. All votes were recorded and presented in real time with the recommendations. Table 2 is a summary of all the recommendations, the level of evidence supporting each recommendation, and the strength of each recommendation.

**Table 2:** Summary of recommendations

Recommendation	Level of evidence and strength of recommendation
***Diagnosis of HAE***	
1. The diagnosis of HAE-1/2 should be made by measuring plasma levels of C4, C1-INH antigen and, when necessary, C1-INH function	High, Strong
2. All individuals with a positive family history should be considered to be at risk of HAE and should be screened as early as possible	Consensus, Strong
***Acute treatment of HAE-1 and HAE-2***	
3. Effective therapy should be used for the acute treatment of attacks of angioedema to reduce duration and severity of attacks	High, Strong
4. Intravenous pdC1-INH is an effective therapy for the acute treatment of attacks	High, Strong
5. Icatibant is an effective therapy for the acute treatment of attacks	High, Strong
6. Ecallantide is an effective therapy for the acute treatment of attacks	High, Strong
7. Intravenous rhC1-INH is an effective therapy for the acute treatment of attacks	High, Strong
8. Attenuated androgens should not be used for the acute treatment of attacks	Low, Strong
9. Tranexamic acid should not be used for the acute treatment of attacks	Low, Strong
10. Frozen plasma could be used for acute treatment of attacks if other recommended therapies are not available	Low, Strong
11. Attacks should be treated early to reduce morbidity (level of evidence: moderate) and mortality (level of evidence: consensus)	Moderate, Strong/Consensus, Strong
12. All attacks of angioedema involving the upper airway are medical emergencies and must be treated immediately	Low, Strong
***Acute treatment and short-term prophylaxis of HAE in pregnant patients***	
13. pdC1-INH is the treatment of choice for angioedema attacks in pregnant HAE-1/2 patients	Consensus, Strong
***Acute treatment of HAE in paediatric patients***	
14. All paediatric patients diagnosed with HAE should have access to acute treatment, including those that are symptom free	Consensus, Strong
15. Intravenous pdC1-INH is an effective therapy for the acute treatment of HAE-1/2 attacks in paediatric patients	Moderate, Strong
16. Icatibant is an effective therapy for the acute treatment of HAE-1/2 attacks in paediatric patients	Consensus, Strong
17. Intravenous rhC1-INH is an effective therapy for the acute treatment of HAE-1/2 attacks in paediatric patients	Consensus, Strong
18. Ecallantide is an effective therapy for the acute treatment of HAE-1/2 attacks in adolescent patients	Consensus, Strong
***Diagnosis of HAE with normal C1-inhibitor***	
19. If the diagnosis of HAE nC1-INH is suspected, a referral should be made to a physician who has expertise with this condition. Testing for gene variants known to be associated with the condition should be performed	Low, Strong
***Acute treatment of HAE with normal C1-inhibitor***	
20. pdC1-INH is an effective therapy for the acute treatment of attacks in patients with HAE nC1-INH	Moderate, Strong
21. Icatibant is an effective therapy for the acute treatment of attacks in patients with HAE nC1-INH	Consensus, Strong
***Short-term prophylaxis***	
22. Short-term prophylaxis should be considered prior to known patient-specific triggers and for any medical, surgical or dental procedures	Low, Strong
23. HAE-specific acute treatment should be available during and after any procedure	Low, Strong
24. Intravenous pdC1-INH should be used for short-term prophylaxis in patients with HAE	Consensus, Strong
***Long-term prophylaxis in HAE-1 and HAE-2***	
25. Long-term prophylaxis may be appropriate for some patients to reduce frequency, duration, and severity of attacks	High, Strong
26. pdC1-INH is an effective therapy for long-term prophylaxis in patients with HAE-1/2	High, Strong
27. Lanadelumab is an effective therapy for long-term prophylaxis in patients with HAE-1/2	High, Strong
28. Subcutaneous C1-INH or lanadelumab should be used as first-line therapy for long-term prophylaxis in patients with HAE-1/2	Consensus, Strong
29. Attenuated androgens and anti-fibrinolytics should not be used as first-line therapy for long-term prophylaxis in patients with HAE-1/2	Consensus, Strong
30. Attenuated androgens are an effective therapy for long-term prophylaxis in some patients with HAE-1/2	Moderate, Strong
31. All patients should have a management plan including immediate access to effective treatment for attacks, even when on prophylaxis	Consensus, Strong
***Long-term prophylaxis in pregnant HAE patients***	
32. When long-term prophylaxis is indicated in pregnancy, pdC1-INH is the treatment of choice	Consensus, Strong
33. Attenuated androgens should not be used during pregnancy or during the breastfeeding period	Consensus, Strong
***Long-term prophylaxis in paediatric HAE patients***	
34. When long-term prophylaxis is indicated in paediatric patients, pdC1-INH is the treatment of choice	Consensus, Strong
35. Androgens should not be used for long-term prophylaxis in paediatric patients	Moderate, Strong
***Self-administration***	
36. All HAE patients should be trained on self-administration of HAE-specific therapies if they are suitable candidates. If patients cannot self-administer therapy, provisions should be made to ensure timely access to all appropriate therapies	Low, Strong
***Approach to individualized therapy***	
37. The decision to start or stop long-term prophylaxis depends on multiple factors and should be made by the patient and an HAE specialist	Consensus, Strong
***Quality of life***	
38. Healthcare providers should routinely assess quality of life in HAE patients using validated instruments in order to optimize HAE management	Consensus, Strong
***Comprehensive care***	
39. Comprehensive care for all patients with HAE should be provided to optimize treatment and outcomes	Consensus, Strong
40. All HAE patients should be informed about HAE patient association(s)	Consensus, Strong
***Registries***	
41. Physicians should participate in an HAE registry and offer patients enrolment	Consensus, Strong

For each topic area, group discussions were recorded and used to inform the clinical considerations for each recommendation.

Prior to the Conference, the Committee determined that open discussion amongst Conference Participants regarding an approach to individualized therapy would be beneficial. For this topic, small round table discussions were facilitated prior to recommendation review and voting, and additional clinical considerations were evaluated.

## Guideline recommendations

### Diagnosis of HAE-1/2

#### Background

The consequences of undiagnosed HAE can be severe. One study demonstrated a mortality rate of 31.4% for undiagnosed HAE patients (n = 63/201) compared to 1.33% in diagnosed patients (n = 7/527) [1]. Without an accurate diagnosis, patients may be unable to access appropriate medications to prevent morbidity and mortality, and may be subject to stigmatization because of their condition. The diagnosis of HAE-1/2 should be based on a thorough history and clinical features supportive of the disease. HAE should be suspected in patients who have recurrent angioedema without concomitant urticaria and also in patients who have recurrent abdominal pain for which no cause is identified, particularly if there is a family history. Healthcare providers should keep in mind that medications known to cause angioedema, such as angiotensin-converting enzyme inhibitors (ACEi) and estrogen-containing oral contraceptives, do not automatically rule out a diagnosis of HAE since these are also associated with angioedema attacks in affected individuals.

In a patient suspected to have HAE-1/2, plasma C4 level is a valuable screening test, with most of those affected having a reduced level between attacks [24] and nearly 100% having a low level during attacks [6, 7]. If C4 is low, further tests can distinguish HAE-1, which has low antigenic C1-INH levels and low functional C1-INH levels, from HAE-2, which has normal antigenic C1-INH levels but low functional C1-INH levels. Results should be confirmed with duplicate laboratory investigations after initial testing. However, HAE-specific treatment should not be delayed while awaiting confirmatory testing.

The Committee affirmed that the diagnosis and management of acquired C1-INH deficiency is not a specific focus of this guideline.
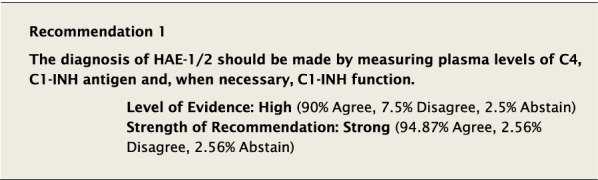


#### Clinical considerations

The availability of assays used to diagnose HAE can vary considerably around the world. Although assays to measure C4 are widely available and relatively inexpensive, C4 level alone should not be used to confirm or rule out a diagnosis of HAE-1/2 [7].

When diagnosing HAE-1/2 in pregnancy, serum C1-INH testing should be interpreted with care as levels of C1-INH can be temporarily low but normalize after delivery in normal pregnancy [25]. The test should be repeated postpartum for confirmation.

Diagnosis in infants can also pose a problem. The C1-INH concentration in umbilical cord blood of healthy neonates is usually lower than that of the normal adult value [26]. Interpretation of C1-INH levels and function can lead to an inaccurate diagnosis in infants less than 12 months old. As such, C1-INH test results should be confirmed after 1 year of age [27]. Given the diagnostic uncertainty of biochemical tests in young children, genetic testing might be a useful option for determining whether a child has inherited HAE-1/2 provided the variant in the affected parent is known.
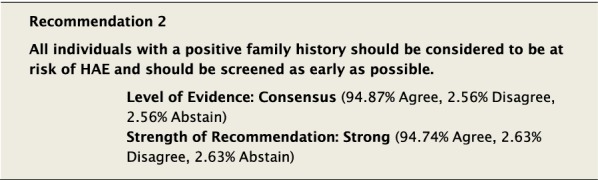


#### Clinical considerations

HAE is an autosomal dominant disease, which gives every offspring of an individual with HAE a 50% chance of inheriting the disease. However, a negative family history does not rule out the possibility that a patient has HAE. Up to 25% of cases are caused by de novo genetic variants [4, 28], meaning that many patients may be affected without a family history of HAE.

### Acute treatment of HAE-1 and HAE-2

#### Background

Attacks of HAE may be spontaneous or precipitated by external stimuli and range from mild to life threatening. The decision to treat an attack depends on many variables and the severity of an attack cannot always be predicted by its earliest manifestations. The aim of treating an attack is to reduce the duration and severity of an attack, to minimize the impact of an attack on the functional ability of the patient, and reduce morbidity and potential mortality.

Despite the increase in available efficacious therapies, some therapies, which have not been demonstrated to be effective in trials, continue to be used to treat attacks due to either historical precedent or lack of awareness.

Ten randomized trials were identified which demonstrated improvement in duration and severity of attacks of HAE-1/2 [29–38]. The therapies studied were plasma-derived C1-INH (pdC1-INH), recombinant human C1-INH (rhC1-INH), icatibant and ecallantide. Table 3 lists the specific agents, their mechanism of action, their licensed indications internationally, the recommended dosages, and important potential adverse reactions. The quality of the evidence for the studies is described under the drug-specific recommendations that follow. We determined this body of evidence to be of high quality, based on the rating of each study using the Cochrane Risk of Bias Tool (see Additional file 1) and the overall consistent effect of therapy on the relevant outcomes (reduction of duration and severity of attacks and effect size).

**Table 3:** Therapies for HAE supported by high level evidence

HAE-specific treatment	Product name and company	Mechanism of action	Approved indications	Dose and route of administration	County licensed and age indications
pdC1-INH	Berinert^®a^ (CSL)	Replaces C1-INH	Acute treatment	20 U/kg intravenous	Australia, Canada, EU, USA (adult and pediatric)
			Pre-procedural	Adults: 1000 U Pediatrics: 15 to 30 U/kg body weight	EU (adult and pediatric)
	Cinryze^®^ (Shire—now part of Takeda)	Replaces C1-INH	Acute treatment	≥ 12 years: 1000 U intravenous 2–11 years: 1000 U (> 25 kg body weight) 500 U (< 25 kg body weight)	Australia (≥ 12 years) EU (≥ 2 years)
			Pre-procedural	≥ 12 years: 1000 U intravenous 2–11 years: 1000 U (> 25 kg body weight) 500 U (< 25 kg body weight)	Australia (≥ 12 years) EU (≥ 2 years)
			Long-term prophylaxis	1000 U intravenous q 3–4 days (6–11 years 500 U q 3–4 days)^b^	Australia, Canada (≥ 12 years) EU, USA (≥ 6 years)
	Haegarda^®^ (CSL)	Replaces C1-INH	Long-term prophylaxis	60 U/kg body weight twice weekly (every 3–4 days)	Australia^c^, Canada, EU^d^, USA (≥ 12 years)
rhC1-INH	Ruconest^®^ (Ruconest)	Replaces C1-INH	Acute treatment	50 U/kg intravenous (< 84 kg); 4200 U intravenous (≥ 84 kg)	EU (adults), USA (adults and adolescents)
Ecallantide	Kalbitor^®^ (Shire—now part of Takeda)	Selective, reversible inhibitor of plasma kallikrein	Acute treatment	30 mg (3 × 10 mg/1 ml) subcutaneous injections	USA (≥ 12 years)
Icatibant	Firazyr^®^ (Shire—now part of Takeda)	Synthetic selective and specific antagonist of bradykinin 2 receptor	Acute treatment	30 mg subcutaneous injection; dose-adjusted for adolescents < 65 kg and children ≥ 2 years^e^	USA (≥ 18 years) Australia, Canada, EU (≥ 2 years)
Lanadelumab	Takhzyro^®^ (Shire—now part of Takeda)	Fully human monoclonal antibody that binds plasma kallikrein and inhibits its proteolytic activity	Long-term prophylaxis	300 mg subcutaneous injection every 2 weeks a dosing interval of 300 mg every 4 weeks may be considered if the patient is well-controlled (e.g., attack free) for more than 6 months	Australia, Canada, EU, USA (≥ 12 years)

Please refer to current country-specific monographs for further details regarding specific indications and listings of adverse events

^a^Berinert 1500 in EU

^b^Dose-adjustment up to 2500 U q3–4 days for ages 12 and above, and up to 1000 U q3–4 days for ages 6-11, based on patient response

^c^Berinert SC in Australia

^d^Berinert 2000/3000 in EU

^e^12 kg to 25 kg: 10 mg (1.0 ml); 26 kg to 40 kg: 15 mg (1.5 ml); 41 kg to 50 kg: 20 mg (2.0 ml); 51 kg to 65 kg: 25 mg (2.5 ml); > 65 kg: 30 mg (3.0 ml)

Based on the Level of Evidence, the potential severity of the outcomes and the low risk of adverse events, we voted for a strong recommendation in favour of the use of effective therapies for the acute treatment of attacks. 
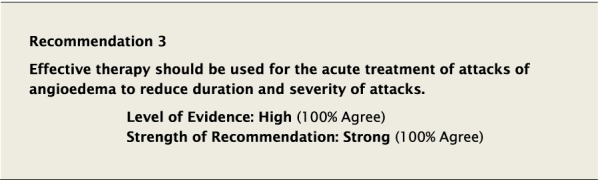


#### Clinical considerations

We emphasized the importance of healthcare professionals only using effective therapies supported by evidence, specifically not using non evidence-based therapies such as antihistamines, corticosteroids and epinephrine, which treat histamine-mediated angioedema. In addition to acute therapy, patients should discontinue and avoid any known triggers such as estrogen-containing oral contraceptives or replacement therapy, dipeptidyl peptidase IV (DPP-IV) inhibitors, neprilysin inhibitors, and ACE inhibitors [39–46].
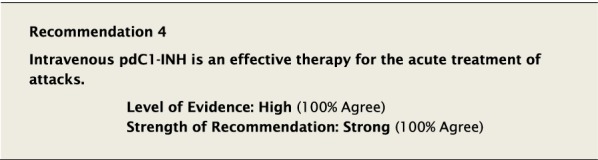


#### Clinical considerations

Plasma-derived (pd) C1-INH is a human blood product. Treatment with pdC1-INH replaces the deficient protein in patients with HAE-1/2. It has been shown to effectively treat attacks of HAE-1/2 in adults and children [31], and is administered either by healthcare professionals or by patients and their caregivers who have been trained in its administration.

The recommended dose for acute treatment of attacks is derived from clinical trials. There have been no head to head trials comparing products so it cannot be concluded that they all have equivalent efficacy. The pdC1-INH products are safe and well tolerated when used as indicated, with no documented transmission of infectious agents.
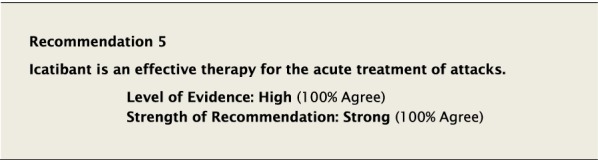


#### Clinical considerations

Bradykinin is a key mediator in inducing angioedema through activation of the bradykinin B2 receptor [8]. Icatibant is a synthetic decapeptide and acts as a selective bradykinin B2 receptor competitive antagonist. It is administered as a single 30 mg subcutaneous (SC) injection. It is generally systemically well tolerated, although 97% of patients experience transient local pain, swelling, and erythema at the injection site.
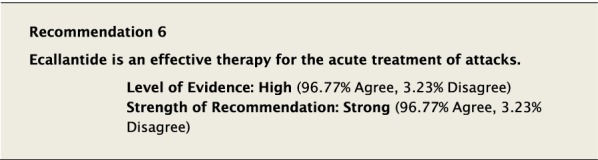


#### Clinical considerations

Plasma kallikrein generates bradykinin through cleavage of high-molecular-weight kininogen [8, 47, 48]. Ecallantide is a 60-amino acid recombinant protein that selectively and reversibly inhibits kallikrein. It is administered as three 10 mg SC injections for a total dose of 30 mg [49]. It has been shown to effectively treat attacks in adolescent and adult patients with HAE-1/2 [35]. Hypersensitivity reactions have been described with this agent in 5% of administrations, of which approximately 50% were possible anaphylactic reactions. It should only be administered by a healthcare practitioner in a location where anaphylaxis can be appropriately managed. 
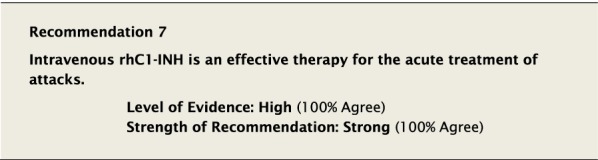


#### Clinical considerations

Recombinant human (rh) C1-INH (conestat-α) is generated in the mammary glands of transgenic rabbits, and is identical to pdC1-INH except for the degree of protein glycosylation [50]. This difference in glycosylation results in shorter plasma mean half-life of the recombinant product [51, 52], however the effect this has on physiologic activity is not known [53]. It has been shown to effectively treat attacks in children 13 years of age and older and adults with HAE-1/2 [37]. Because of an isolated anaphylactic reaction after administration of rhC1-INH to a rabbit-allergic person, those known or suspected of having a rabbit allergy should not receive rhC1-INH.
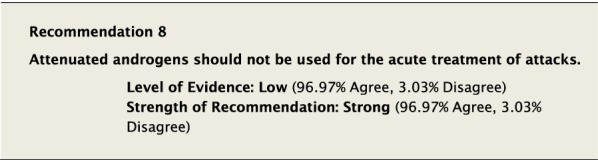
 
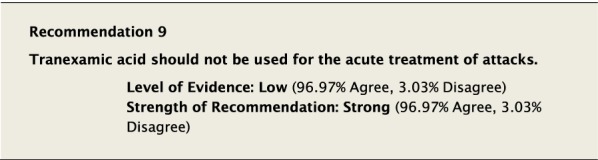


#### Clinical considerations

Attenuated androgens such as the 17 α-alkylated anabolic androgen danazol and anti-fibrinolytic drugs such as tranexamic acid have not been shown to be efficacious in the acute treatment of attacks of HAE-1/2. Given the lack of evidence for these agents in the acute treatment of HAE, we strongly agreed that they should not be used for the treatment of acute HAE attacks.
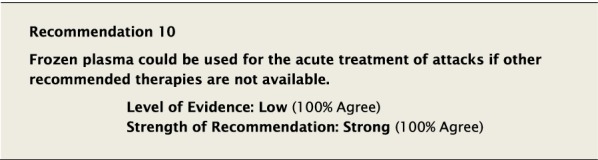


#### Clinical considerations

Frozen plasma (FP) is a blood product, which contains C1-INH in association with other plasma proteins. FP is not as safe as solvent detergent plasma (SDP) with respect to pathogen inactivation, and the level of evidence that FP is effective in the acute treatment of attacks of HAE-1/2 is low. It also contains potential substrates for the generation of additional bradykinin and in theory could worsen attacks of angioedema. There may be a significant delay in getting FP and/or SDP in a timely manner—in some cases up to 24 h. Therefore, we felt strongly that frozen plasma products, although potentially beneficial, should only be used if other recommended therapies are not available, and that every effort should be made to ensure timely and appropriate therapy for HAE attacks [54, 55]. A decision to use FP will depend on local and patient factors and will require risk/benefit assessment of the respective merits of local FP treatment versus symptomatic relief only. Thus the threshold for treating when only FP is available would usually be limited to life-threatening or severely painful attacks.
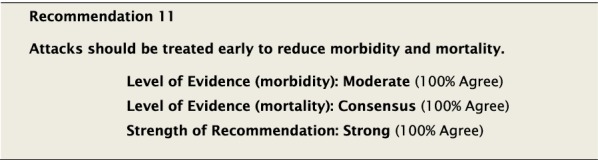


#### Clinical considerations

Early treatment leads to more rapid symptom resolution. Observational studies have suggested that early treatment can be efficacious in reducing the duration of an HAE attack in some patients [56–62]. Therefore, despite the absence of a high level of evidence, we strongly endorsed early treatment in an attempt to reduce morbidity and likely mortality. Interestingly, early treatment with C1-INH might also reduce the overall severity of an attack in addition to reducing the time to symptom relief [63]. Because of the potential barriers in accessing therapy in a timely manner, patients should be trained on how to self-administer therapies appropriate for the acute treatment of HAE attacks. If patients are not able to self-administer, efforts should be made to ensure that this therapy is made available to them without a significant delay (see Recommendation #36 for further details).
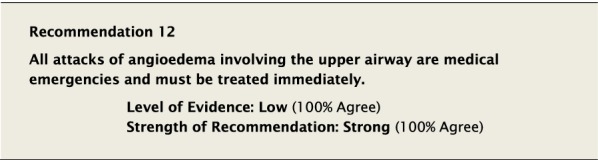


#### Clinical considerations

Attacks of HAE are unpredictable and potentially life threatening. Mortality due to laryngeal angioedema is well recognized [1]. All attacks of laryngeal angioedema should be considered medical emergencies, and therapies that have been shown to be effective in the treatment of HAE should be readily available and given immediately. It is also recommended that all patients with laryngeal edema, even following self-therapy, be assessed in the emergency department in the event that the angioedema does not respond to therapy and expertise in airway management is required [64].

### Acute treatment and short-term prophylaxis of HAE in pregnant patients

#### Background

The gynaecologic and obstetrical management of patients with HAE-1/2 presents a unique challenge to healthcare providers. Pregnancy in particular may worsen, improve, or have no effect on angioedema attacks [65–68], and that effect may change from pregnancy to pregnancy [68]. At present there doesn’t appear to be a clear trend of whether symptoms are more severe in any specific trimester [67, 69]. Vaginal delivery does not appear to be a trigger [65–68, 70] suggesting that women can forego STP for an anticipated uncomplicated vaginal delivery, although acute treatment should always be readily available. STP is recommended for C-sections or intra-partum instrumentation (see “Short-Term Prophylaxis” section for further details). Pregnant HAE patients should be closely monitored by an HAE expert in conjunction with a team of relevant medical professionals. Due to ethical reasons, there are no randomized controlled trial data assessing the efficacy and safety of medications used to treat attacks in pregnant HAE patients, although many cases have been documented. Guideline Authors recommended that healthcare professionals register the treatment of all their pregnant HAE patients in order to contribute to the body of literature on treatment outcomes in this group.

The Committee affirmed that lactation and menstruation in patients with HAE were not specifically addressed in this guideline.
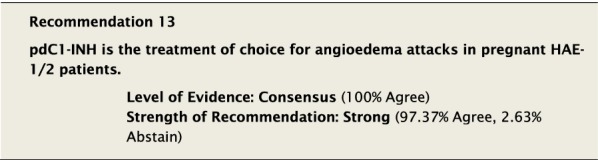


#### Clinical considerations

We unanimously agreed that the level of evidence for the use of pdC1-INH to treat acute HAE attacks in pregnancy was consensus, based on several case reports and observational studies [66–69, 71–74]. However, we strongly endorsed pdC1-INH as the treatment of choice in an effort to reduce morbidity and mortality in pregnant HAE-1/2 patients. For the same reason, despite specific evidence, we also strongly recommended using pdC1-INH for angioedema attacks in pregnant HAE nC1-INH patients. The licensed indications and recommended dosing of pdC1-INH for the acute treatment of attacks are listed in Table 3. Icatibant [75] or rhC1-INH [76] may be used in the case of life-threatening attacks during pregnancy when pdC1-NH is not available or has not been efficacious for a particular patient.

### Acute treatment of HAE in paediatric patients

#### Background

This section covers the acute treatment of paediatric patients (children ≤ 12 years of age and adolescents 12–17 years of age), and will focus solely on HAE-1/2. While HAE can present at any age, the reported age of onset of attacks varies from 4.4 to 18 years with the mean age of first attack at 10 years [77]. Disease presentation in infancy is uncommon, but it is possible that abdominal symptoms of HAE are overlooked in infants. Abdominal discomfort and pain are common complaints in childhood and may easily be mistaken to have other causes in this population [78–82].
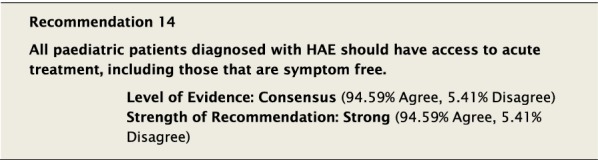


#### Clinical considerations

Symptom-free is in reference to those patients who have been diagnosed with inherited C1-inhibitor deficiency, but who have yet to demonstrate any symptoms of the disease. Due to the risks associated with the disease, all diagnosed patients should have ready access to acute treatment either on site or at a nearby medical facility. The following are drug-specific recommendations for the acute treatment of HAE attacks in paediatric patients.
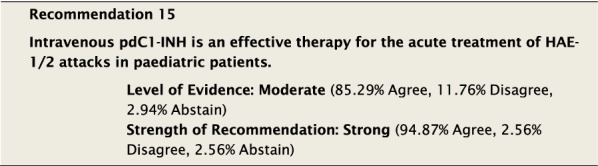


#### Clinical considerations

When children are treated with pdC1-INH for HAE attacks, responses are consistent with that of adults [83]. Studies have demonstrated that the intervention is safe and well tolerated [31] in the paediatric population and effective in reducing time to symptom relief [32, 84, 85]. Also similar to adults, data suggest that early treatment with pdC1-INH leads to more rapid symptom resolution [59, 62]. Dosing for pdC1-INH is 20 units (U)/kg IV Berinert^®^ (CSL) [31, 83, 86, 87], 500 U IV Cinryze^®^ (Takeda) for children 10–25 kg, or 1000 U IV Cinryze^®^ for children > 25 kg [88–90].
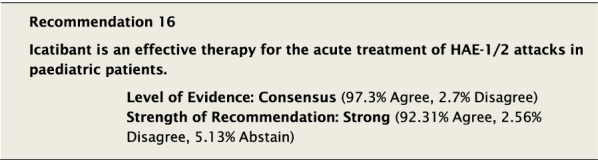


#### Clinical considerations

Icatibant has been approved to treat patients ≥ 2 years of age in some countries (see Table 3) [91]. Depending on the age of the patient, the single SC dose of 0.4 mg/kg (to a maximum dose of 30 mg) injected into the abdomen can be self-administered, or given by a caregiver particularly in children. It does not require intravenous access, which can be challenging in paediatric patients [91–93].
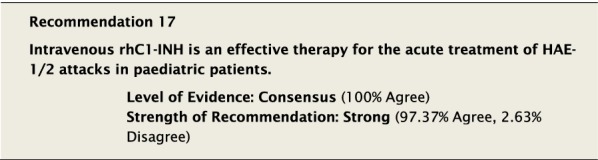


#### Clinical considerations

Intravenous rhC1-INH is a recombinant form of C1-INH that has been studied in adolescents and adults for the acute treatment of HAE attacks [37]. Dosing is weight based, 50 U/kg (unless a patient is ≥ 84 kg, then the dose is 4200 U), and delivered intravenously [94].
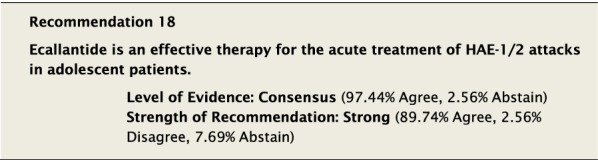


#### Clinical considerations

Ecallantide has been studied and approved for use in adolescents ≥ 12 year of age in the USA based on data from 4 clinical studies [49, 95]. This small pooled data set showed efficacy in children as young as 10 years of age. Ecallantide is administered as 3 SC injections for a total of 30 mg [49], but cannot be self-administered.

### Diagnosis of HAE with normal C1-inhibitor

#### Background

The presence of HAE nC1-INH was first reported in Canada and Germany by Binkley and Bork respectively in 2000 [96, 97]. As of 2018, there were over 200 identified families with the disease worldwide, however the true prevalence remains unknown and there is significant variation in prevalence between countries. HAE nC1-INH can be further subdivided by causative variant affecting coagulation factor XII (HAE-FXII), angiopoietin-1 (HAE-ANGPT), plasminogen (HAE-PLG), and unknown (HAE-U) [11].
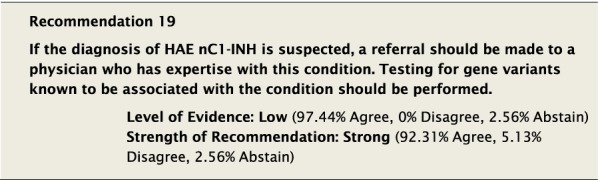


#### Clinical considerations

We recommended that the diagnosis of HAE nC1-INH be based on clinical evaluation by an expert physician. Criteria for the diagnosis of HAE nC1-INH include: (1) a history of recurrent angioedema in the absence of concomitant hives or use of medication known to cause angioedema; (2) documented normal or near normal C4, C1-INH antigen, and C1-INH function; and (3) either a genetic variant associated with the disease, or a family history of angioedema and documented lack of efficacy of chronic high-dose antihistamine therapy [16]. Healthcare providers should also have a strong index of suspicion for HAE nC1-INH if a patient presents with the above criteria and has failed corticosteroids and/or a trial of omalizumab. Testing for gene variants known to be associated with the condition should be performed where possible. The Guideline Authors affirmed that these diagnostic criteria are based on ideal feasibility and availability of the above tests and should not be considered absolute requirements in order to make the diagnosis of HAE nC1-INH.

### Acute treatment of HAE with normal C1-inhibitor

#### Background

HAE nC1-INH is a rare disease that can be a challenge to diagnose with certainty as discussed above. This creates a unique set of challenges for patients since treatments are hard to access without a diagnosis. Patients may also be stigmatized due to a lack of understanding of their condition especially if they are presenting regularly to the emergency department with angioedema attacks, but have documented normal C1-INH. It has been suggested, without confirmatory evidence, that bradykinin may play a role in the pathogenesis, leading to speculation that therapies used for HAE-1/2 may be beneficial [98]. There is also indirect evidence that antihistamine therapy is not effective in this patient group [99].
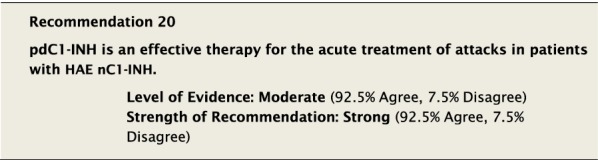
 
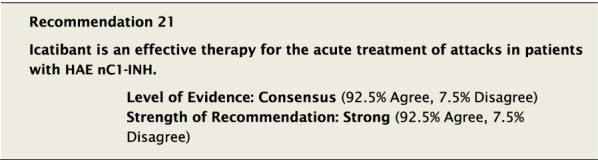


#### Clinical considerations

Guideline Authors felt they could recommend trials of specific therapies that have proved effective in some cases because, while there have been neither significant case series nor controlled clinical trials studying therapeutic intervention for attacks in the HAE nC1-INH population, these attacks are often severe and may be life-threatening. In addition to acute therapy, patients should discontinue and avoid any known triggers such as estrogen-containing oral contraceptives or replacement therapy, DPP-IV inhibitors, neprilysin inhibitors, and ACE inhibitors [39–46].

Intravenous pdC1-INH has efficacy in reducing the duration and intensity of attacks of angioedema in patients with HAE nC1-INH (as shown by non-controlled, retrospective studies on small case series recruited with non-predetermined homogeneous criteria) despite the fact that the pathogenesis of the angioedema, by definition, is not caused by a deficiency in C1-INH [15, 40, 41, 45].

With the same limitations as for pdC1-INH, there is evidence that blocking bradykinin-2 receptors with icatibant is an effective intervention for treating attacks in various body sites in the majority of HAE nC1-INH patients [15, 39, 100].

The Committee affirmed that these recommendations generalize to all HAE nC1-INH patients and are not subdivided by causative variant in this guideline.

### Short-term prophylaxis

#### Background

Short-term prophylaxis (STP) refers to the practice of treating patients to reduce the risk of associated and consequent morbidity and mortality during a period of time when there may be an increased risk of having an angioedema attack.

It is well recognized that physical trauma, as can occur during medical and dental procedures, can induce episodes of angioedema [101–103]. Upper airway manipulation, including during dental surgery and intubation, is particularly high risk due to its association with upper airway swelling. However, even minor procedures can precipitate angioedema, and the ability to predict when this may occur cannot be made with certainty. Attacks can occur anywhere from hours to several days after a procedure [101]. Healthcare providers should educate their patients—regardless of whether they received STP—about the possibility of angioedema attacks happening within 72 hours post-procedure.

It is also thought that other causes, such as emotional stressors can precipitate attacks. Individual patients may also be aware of specific triggers that have been known to trigger their attacks. Despite these observations, there are no controlled clinical trials in this area, and data come from personal experience, retrospective reviews, and surveys [101, 102, 104, 105].
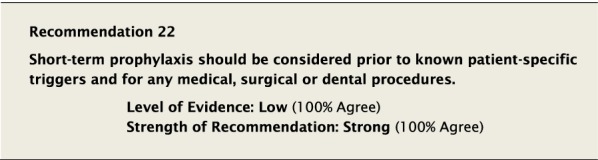
 
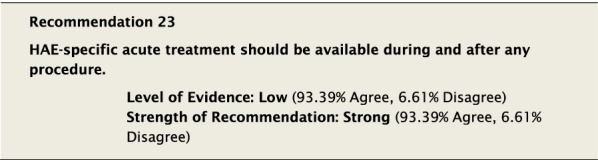


#### Clinical considerations

There was extensive discussion as to when STP should be used, and consideration was given to the development of a list of high- and low-risk procedures in this context. However, there is a lack of data regarding the specific risk associated with each of a wide range of medical, surgical and dental procedures, and that STP should be considered for some. One study assessed the risk of angioedema following surgery without pre-procedural prophylaxis as 5–30%, irrespective of type and extent of surgery [101]. Based on this, and our inability to link the risk of an attack to a specific procedure [101, 102], it was felt that STP should at least be considered for procedures that are near the upper airway, or cause trauma, or are known patient-specific triggers. This recommendation was intended to remain broad in its scope, as the risk of appropriate STP would likely be minimal compared to any real or perceived risk of not using STP when felt necessary. Ultimately, the decision of whether or not to use STP should involve both the patient and their HAE healthcare provider and may depend on several factors including the degree of physical trauma involved and whether or not the patient has had an attack previously under similar circumstances. Dental extraction, for example, is likely higher risk for inducing angioedema than dental cleaning or cavity restoration, and healthcare providers may choose to not use prophylaxis for perceived low-risk procedures unless similar procedures have precipitated attacks in the past. If the decision is made not to administer STP, all patients should have two doses of appropriate on-demand therapy immediately available as per Recommendation #23. Even those that receive STP should have two on-demand treatments available. What is not known from the current data is how many patients have been denied or have chosen not to pursue necessary procedures due to perceived risks, or have not been offered STP. Ensuring access to STP may help mitigate the risk associated with procedures and enable patients to seek and receive the care they need [106].
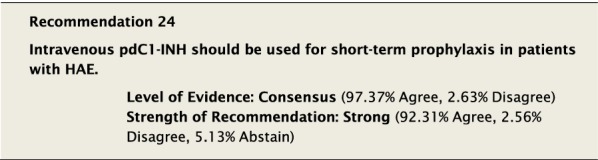


Pre-procedural prophylaxis with pdC1-INH concentrate is recommended at a dose of 20 U/kg IV within an hour before a procedure. In one study, patients had about a 30% risk with no prophylaxis and a 15% risk with 500 U of pdC1-INH, which was reduced to about a 5% risk at 1000 U [102]. With 20 U/kg the average-sized person would be receiving 1500 U, which hopefully will reduce the 5% risk of an attack following a procedure. Furthermore, given that breakthrough attacks have occurred even with prophylactic pdC1-INH concentrates at 1000 U, at least two additional treatments for attacks should be available. In Europe, Cinryze^®^ is licensed in adults for 1000 U to be given within 24 h of an anticipated procedure or Berinert^®^ 1000 U within 6 h. The European paediatric licensing is dosed by weight with 500 U of Cinryze^®^ for children 10–25 kg (within 24 h of an anticipated procedure) or 15–30 U/kg of Berinert^®^ (within 6 h) (see Table 3).

Attenuated androgens or frozen plasma may be considered for STP when pdC1-INH is not available and particularly if HAE-specific acute treatments are not available. When androgens are chosen for STP, danazol can be considered starting 5 days before the anticipated procedure or trigger, and continuing 2–3 days after the anticipated trigger (danazol 2.5 to 10 mg/kg/day, maximum 600 mg/day) [9]. Disadvantages with androgen therapy include perceived inferior efficacy to pdC1-INH concentrate. Attenuated androgens are not suitable in pregnancy or during breastfeeding, and a pregnancy test should be considered before initiation of therapy with androgens. Frequent short-term uses may be associated with similar effects seen with long-term androgen use as discussed in “Long-Term Prophylaxis of HAE-1 and HAE-2”. The optimal dose of frozen plasma for STP has not been determined but, based on cases in the literature, it is typically given as 2 U in adults and 10 mL/kg in children 1 to 2 h prior to a procedure [55, 107–109].

Anti-fibrinolytic agents such as tranexamic acid have been used for STP with suggested dosages of 25 mg/kg 2–3 times daily to a maximum of 3–6 g per day, 5 days before and 2–5 days after a procedure or anticipated trigger. The efficacy for prevention of attacks, however, is unknown and this agent should be used only if other therapies are not available.

Lanadelumab (Takeda), a humanized monoclonal antibody against kallikrein, takes approximately 70 days to reach a steady state concentration [110], and is therefore not recommended for STP. Similarly, there is a delay in reaching the therapeutic steady state for a week or two with subcutaneous C1-INH suggesting it should not be used as STP. Data are lacking as to whether STP is necessary if patients are symptom free with either lanadelumab or subcutaneous C1-INH, and until these data are available STP, if indicated, should be used when prescribing either for LTP.

In general, pregnant HAE patients do not appear to require routine STP for uncomplicated vaginal deliveries [65, 67, 68, 70]. However, there are certain instances where STP could be considered prior to vaginal delivery including a history of severe HAE attacks, frequent attacks during the third trimester, or a history of genital edema secondary to mechanical trauma [66, 70, 111–113]. STP is recommended in the case of a C-section or intra-partum instrumentation, and may need to be repeated, subject to drug half-life, if delivery hasn’t happened within a certain time period [66, 68]. As with other procedures, two doses of on-demand therapy should be available in case of an HAE attack.

### Long-term prophylaxis in HAE-1 and HAE-2

#### Background

Long-term prophylaxis (LTP) refers to the use of ongoing, regular treatment to prevent attacks of HAE when on-demand treatment does not sufficiently meet patient treatment requirements as discussed below in “Approach to individualized therapy” section. Prophylactic therapy may be considered for patients with recurrent episodes of angioedema to reduce the frequency, duration and severity of attacks. The specifics of when to consider and when to initiate LTP are discussed below. 
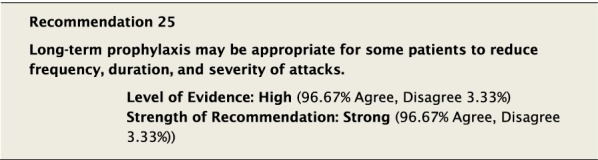


#### Clinical considerations

The aim of LTP is to reduce the frequency and/or severity of attacks of HAE and minimize the impact of HAE on QoL, thereby enabling patients to live normal lives. Some patients may be candidates for long-term therapy, and the benefits and risks associated with such treatments should be explored to optimize patient care. It is important to remember that no prophylactic regimen has been associated with the complete elimination of angioedema. Therefore, despite being on prophylaxis, all patients should be equipped to treat angioedema attacks in a manner consistent with Recommendation #3, and an acute treatment plan should be agreed to between patient and physician.
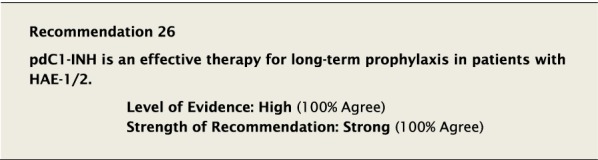


#### Clinical considerations

Controlled clinical trials have demonstrated that both IV and SC pdC1-INH used for prophylaxis in HAE-1/2 reduces the number, duration, and severity of attacks of angioedema [36, 38, 114].

C1-inhibitor prophylaxis has traditionally been given intravenously [38]. More recent trials have shown higher levels of efficacy when C1-inhibitor is given as a higher dose subcutaneously. The subcutaneous route also reduces the inconvenience and medicalization associated with the intravenous route, and avoids hazards of repeated venipuncture and indwelling catheters [115], further improving QoL [116]. However, direct comparison between the IV and SC routes has not been subject to formal trial. 
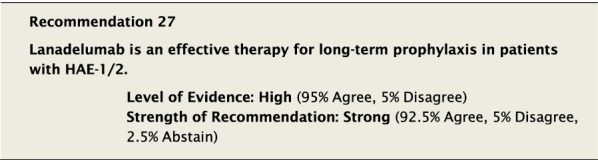


#### Clinical considerations

Lanadelumab is a subcutaneously injectable, fully humanized, anti-active plasma kallikrein monoclonal antibody (IgG1/κ-light chain). It is administered as 300 mg every 2 weeks, however a dosing interval of 300 mg every 4 weeks may be considered if a patient is well controlled (e.g., attack free) for more than 6 months [110]. 
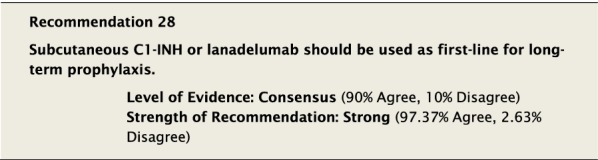


#### Clinical considerations

Although there have not been any head-to-head comparisons of long-term prophylactic agents, hence a consensus level of evidence for efficacy, we strongly agreed that either subcutaneous pdC1-INH or lanadelumab are appropriate as first-line LTP.
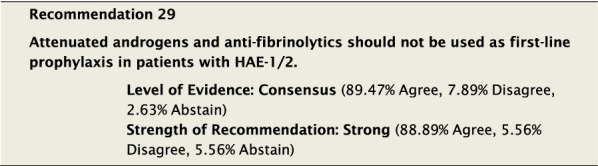
 
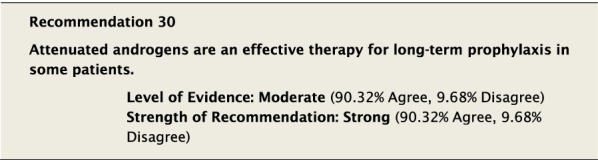


#### Clinical considerations

Considerations when deciding to start prophylaxis are discussed below, in “Approach to individualized therapy” section. The decision to start LTP should be based on the efficacy of the therapy, its side effects and safety profile, and the patient’s preference. Although androgens and anti-fibrinolytics are not recommended as first line, these agents may be considered for LTP in those patients who have already obtained benefit from their use or who have difficulty obtaining first-line options. It should not be necessary for patients to fail other long-term prophylaxis therapies, such as androgens and anti-fibrinolytics, before using pdC1-INH or lanadelumab.

Controlled trials and observational studies have demonstrated that treatment with 17 α-alkylated anabolic androgens, such as danazol, reduces the frequency and severity of HAE attacks [117–122]. Although one of the trials was a randomized controlled trial, the level of evidence for the trial was not considered high as there were insufficient details on funding, sequence generation, and outcome reporting [120]. Historically, many patients have been controlled with androgen therapy and their use in some patients may be acceptable provided that the lowest effective dose is used to achieve efficacy and minimize adverse events. Expert opinion suggests the optimal dose for danazol, to minimize adverse events, is ≤ 200 mg/day [9, 98].

Androgens can affect serum lipid levels, can be hepatotoxic resulting in hepatitis, and have been associated with hepatocellular adenoma and, in very rare cases, carcinoma [118, 123, 124]. It is recommended that all patients on androgen therapy be monitored for hypertension and have a complete blood count, liver enzymes, urinalysis, serum α-fetoprotein, creatine phosphokinase and lipid profile performed every 6 months, and an annual liver ultrasound [17].

Virilising effects of androgen therapy can occur and include menstrual irregularities, masculinization, irreversible voice alteration, and hirsutism. Psychological side effects include emotional irritability and lability, aggressive behaviour and depression. Androgens interact with several medications. They are contraindicated in pregnancy and lactation, before puberty, and in patients with androgen-dependent malignancy and hepatitis [123, 124]. Patients need to be made aware of these side effects when considering and while on androgen therapy, and physicians should carefully consider the risks and benefits for the particular patient.

There is a moderate level of evidence showing the benefit of the anti-fibrinolytic agent tranexamic acid as an LTP agent. This benefit was demonstrated in a randomized placebo-controlled trial with 18 subjects ≥ 12 years taking 1 g of tranexamic acid three times a day [125], and a double-blind crossover study of ε-amino-caproic acid in 9 patients aged 7 to 40 years [126]. These data suggested that anti-fibrinolytic agents could be useful for LTP for HAE-1/2. However, their role in current LTP was felt to be justified only in certain patient groups due to the lack of efficacy and the potential side effects at the dosage studied. The recommended dosage for tranexamic acid is 30–50 mg/kg daily divided in 2 or 3 doses to a maximum of 6 g per day.
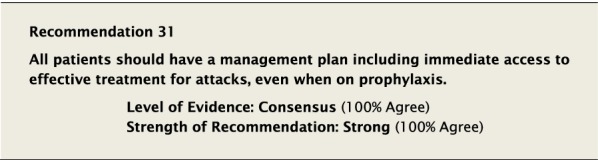


#### Clinical considerations

Since no LTP therapy completely eliminates the risk of attacks, all patients should have access to at least two doses of on-demand therapy and patient competency to administer such therapies should be routinely assessed. Effective treatment for attacks should also be sufficient to provide patients with enough time to access an urgent care centre.

### Long-term prophylaxis in pregnant HAE patients

#### Background

When making decisions regarding LTP for pregnant patients, healthcare providers need to consider efficacy and safety of treatment for mother and infant throughout pregnancy, labour and delivery, as well as during the breastfeeding period. Given the rarity of HAE combined with the ethics of enrolling pregnant patients in clinical trials, it is unlikely there will be any placebo-controlled interventional studies assessing LTP treatment options prospectively in pregnant patients with HAE. The available evidence comes from observational studies, case reports, retrospective reviews, questionnaires, and expert opinion.
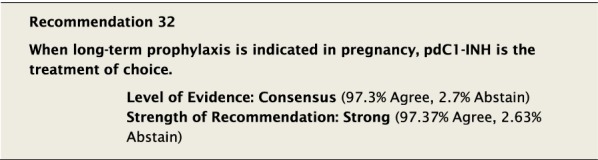


#### Clinical considerations

The data from observational studies [69, 127] and retrospective reviews [67, 68] demonstrated that pdC1-INH was generally safe and not associated with any neonatal abnormalities or treatment-related adverse events during the study periods. Although the data were not of high quality, we strongly recommended pdC1-INH when LTP is required in pregnancy.
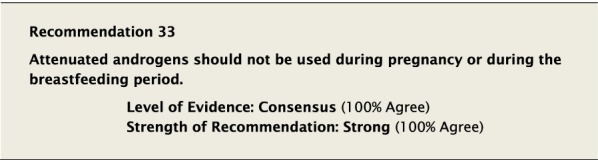


#### Clinical considerations

Androgens are contraindicated during pregnancy as these drugs can have significant effects on the normal development of the fetus, including masculinization. Potential effects on the female fetus include clitoral hypertrophy, labial fusion, urogenital sinus defect, vaginal atresia, and ambiguous genitalia [128–130].

### Long-term prophylaxis in paediatric HAE patients

#### Background

Long-term prophylaxis in the paediatric population needs to be flexible in order to accommodate changes in a patient’s hormones, stressors, and lifestyle [89]. Currently, there are limited data showing the use of newer, specific therapies for routine prophylaxis in children. 
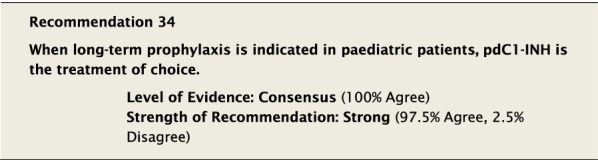


#### Clinical considerations

The clinical studies assessing the use of prophylactic pdC1-INH in children have been of small sample size [84, 85, 131–134]. Pooled data from an RCT and its open-label extension study demonstrated that pdC1-INH was effective and well tolerated for routine prophylaxis in children with HAE. Patients received IV infusions of pdC1-INH 1000 U (500 U for children ages 6 to 11) or placebo every 3 to 4 days. During the placebo-controlled pivotal trial, pdC1-INH reduced the number of angioedema attacks by nearly twofold (n = 4). During the open-label extension, pdC1-INH significantly decreased the pre-enrolment median monthly attack rate (n = 23). Adverse events during the studies were minimal (1 patient with pyrexia in the pivotal trial, and 1 patient with headache and nausea and another with infusion-site erythema considered related to pdC1-INH in the open-label extension) [85]. Lanadelumab and SC pdC1-INH are indicated for routine prevention of recurrent attacks of HAE in patients aged 12 years and older (see Table 3).
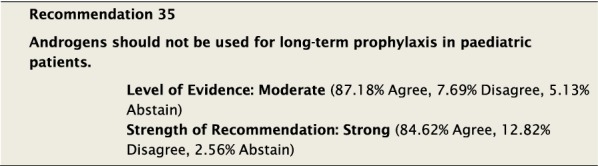


#### Clinical considerations

Androgens are known to cause premature closure of the epiphyses [135, 136], among other significant side effects, and are therefore contraindicated as LTP in the paediatric population before Tanner stage 5. However due to their efficacy, as described above, and in the absence of other available options, androgens may be considered once patients have completed puberty. If androgen use is necessary, paediatric patients should start at the lowest effective dose. They should have regular monitoring for side effects.

Anti-fibrinolytics cannot be recommended for LTP in the paediatric population due to the lack of evidence. Where they have been studied in children, they have shown limited efficacy [133].

Similar to adults, paediatric patients should not be required to fail other non-specific therapies, such as androgens or anti-fibrinolytics, before proceeding to more specific LTP agents.

### Long-term prophylaxis in HAE with normal C1-inhibitor

#### Background

Patients with HAE nC1-INH share similar clinical characteristics with HAE-1/2 patients, including the risk of random, unpredictable attacks of debilitating and potentially life-threatening angioedema [99]. These similarities have led to speculation that treatments used for LTP for HAE-1/2 may be beneficial for patients with HAE nC1-INH. However, due to the lack of data, a recommendation for this intervention could not be made. Guideline Authors felt strongly that more data are needed in this area and that appropriate trials should be done to help guide future treatment recommendations.

#### Clinical considerations

We did not reach consensus on the proposed recommendation for the use of progestins or tranexamic acid for LTP in patients with HAE nC1-INH. There is some evidence that progestins, anti-fibrinolytics and attenuated androgens may be efficacious in patients with HAE nC1-INH [45, 137]. Although some European countries have shown progestins to be effective, the same high doses used in Europe are not available in all countries [66]. Nevertheless, we wanted to re-iterate the importance of avoiding known triggers of angioedema such as estrogen-containing oral contraceptives or replacement therapy, DPP-IV inhibitors, neprilysin inhibitors, and ACE inhibitors [39–46].

### Self-administration

#### Background

Self-administration refers to the treatment of patients outside of a healthcare facility either by the patients themselves or by a trained caregiver. The recognition and support of self-administration as treatment for HAE go back to the first international consensus document on HAE in 2003, and it has been repeatedly recommended in subsequent consensus statements and guidelines [9, 17, 138]. It has been shown to be a safe and convenient option for patients, allows for early treatment, and may reduce the overall treatment costs of this group when compared to hospital-based therapy [139, 140]. However, despite the demonstrated benefits of self-administration and on-demand therapy in terms of efficacy and improved QoL, an online survey done in the USA revealed that only 8.1% of treating physicians had patients who self-treated and only 3.5% received home healthcare-assisted administration [141, 142]. Although specific data in Canada is lacking, there is little reason to believe it would differ significantly from these findings. Self-administration of blood products for rare blood disorders is not without precedent, and has been the cornerstone of effective therapy for haemophilia for more than three decades [143].

Treatment is more efficacious when attacks are treated early [144]. Evidence has shown that the earlier an attack is treated the sooner it resolves [35, 57, 145, 146]. The ability to treat an attack early depends on reducing the number of steps required between recognition of an attack that requires treatment and implementation of effective therapy. Obligating patients to travel to a healthcare facility to receive a therapy, which has been shown to be effective when administered at home or outside of a healthcare facility, adds to the delay in receiving treatment [2] and may result in many attacks not being treated. Patients may also face difficulties in accessing treatment if local healthcare facilities are unfamiliar with this condition. All therapies should be available to all HAE patients worldwide, and home- and self-administration are preferred because they reduce morbidity, absenteeism, cost, disease burden and potentially mortality, as well as improve QoL [17, 145, 147].
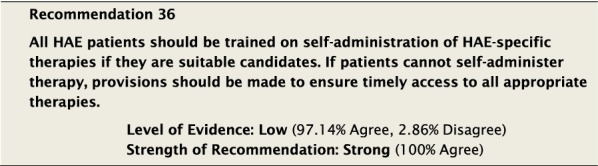


#### Clinical considerations

Although the level of evidence was low for the recommendation that all patients should be trained on self-administration of HAE-specific therapies if they are suitable candidates, it was unanimously considered a strong recommendation. This is consistent with prior consensus statements and guidelines [18, 19, 148]. The importance of early therapy should not be underestimated, and barriers that affect its implementation should be removed. Geographic and regional disparities in care are known to exist, and self-administration of therapy will remove these. Although, intravenous pd-C1INH requires special considerations including product tracking and patient training, the use of blood products for self-administration is not unique. Haemophilia self-administration programs, which are similar, have been implemented and have been shown to be effective [143, 149]. Another example is self-administration of subcutaneous immunoglobulin for patients with primary immunodeficiency disease [150, 151]. Recent licensing of subcutaneously administered therapies will further simplify self-administration [114, 152, 153].

Although not all patients will be suitable candidates for self-administered therapy, the option should be considered in the overall care plan for HAE patients. If patients are considered appropriate and willing to learn self-administered therapy, they should agree to specific criteria as outlined in previously published international home-therapy guidelines [154, 155]. With self-administered therapy, patients need to be regularly monitored to ensure appropriate control of their symptoms, compliance, and competency. This is discussed further in “Approach to individualized therapy” section below.

### Approach to individualized therapy

#### Background

HAE is a dynamic chronic disease, and attacks of angioedema can vary in frequency and severity over the patient’s lifetime. This variability makes it important for patients to be evaluated regularly to ensure that therapy is appropriate, used correctly, and that side effects of therapies are being minimized. A recently published document outlines an approach to monitoring attack frequency and severity [148].

Perhaps one of the most challenging areas in patient treatment is deciding when to start or stop LTP therapy. Although guidelines exist on which agents to use when starting LTP, there is no evidence comparing the use of LTP to acute on-demand therapy regarding benefit and risk. In the absence of such evidence, given the clinical importance of this therapeutic approach, the Committee attempted to determine which variables should be considered when trying to decide when to start or stop LTP.
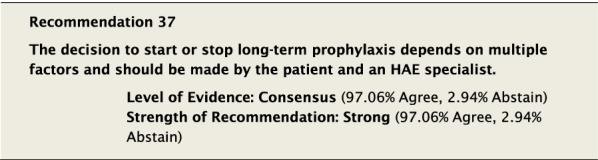


#### Clinical considerations

There was considerable discussion regarding factors that should be considered when deciding to start LTP. It was generally agreed that key considerations in making the decision included the efficacy of on-demand therapy to control the severity and frequency of attacks. Although in the past some consensus documents have tried to define the number and severity of attacks as a reference point to consider when to start LTP [10], there was significant concern about the arbitrary nature by which this would be defined. This approach might lead to denying LTP to patients whose QoL is impacted despite not meeting specific predefined criteria. It was felt that although the frequency of attacks is important, it is only one among many factors that should be considered, along with: severity of previous attacks, how readily patients can access emergency treatment, their ability to administer on-demand therapy, and impact on QoL.

Although the aim of LTP is to reduce the number and severity of attacks, it does not completely eliminate the risk. Patients must be aware that starting LTP does not mean that they will no longer have attacks and that attacks which occur can still be fatal. All patients must have a plan to treat attacks on demand despite being on LTP therapy. All patients must be monitored to ensure that LTP is efficacious and that side effects are being recorded [148].

When starting LTP it is important to understand and emphasize that LTP is not necessarily a lifelong therapy and that treatment needs ongoing re-evaluation. It may be helpful to try to objectively define patient expectations when starting LTP. Part of the monitoring process should be to examine these goals and ensure they are being met.

The decision to stop LTP also generated significant discussion. All Conference Participants felt LTP with androgens should be stopped immediately if a patient became pregnant or was breastfeeding. Reducing or stopping LTP could be considered if the patient has been stable with no evidence of breakthrough attacks of angioedema for a protracted period of time, though no specific guidance can be provided on a specific duration of symptom control and the decision must involve the patient. If the decision to reduce or stop LTP is made, all patients must ensure that they have ongoing access to the administration of appropriate on-demand therapy for attacks as is consistent with Recommendation #36. All members of the patient’s comprehensive care team should be aware of the plan to reduce or stop LTP in case complications arise.

When stopping LTP with attenuated androgens or anti-fibrinolytics, the majority of Conference Participants agreed that a gradual taper is recommended, if the patient is not pregnant, while monitoring the frequency of and the impact on the patient’s QoL. When stopping LTP with pdC1-INH or lanadelumab it was felt it could either be stopped abruptly or the frequency of administration decreased, while monitoring the patient’s response.

The Committee unanimously agreed that the decision to start or stop LTP should be made jointly by the patient and an HAE specialist. The patient needs to be informed of the risks and benefits of all therapies, as discussed in the relevant sections above, to enable making an informed decision. Additionally, long-term effects on vein health need to be taken into account when considering repeated IV infusions.

### Quality of life

#### Background

The Constitution of the World Health Organization (WHO) defines health as, “A state of complete physical, mental, and social well-being not merely the absence of disease”. It follows that the measurement of health and the effects of healthcare must include not only changes in the frequency and severity of diseases, but also an estimation of well-being and disease-specific health-related quality of life (HRQoL). The impact of HAE on a person’s HRQoL can be considerable. A survey done in the USA in 2004 revealed that 85% of patients were afraid of sudden closure of their airway, 75% experienced intolerable pain, and 53% were concerned about transmitting HAE to their offspring [156]. Another study of 457 HAE patients from the USA reported significantly poorer HRQoL versus population norms. [157]. Productivity was also markedly impaired, including 34% overall work impairment. Because of their most recent HAE attack, workers lost a mean of 3.3 days and students lost a mean of 1.9 days. A Swedish registry of HAE patients documented missed days from work and school [158]. A multicentre European Study recorded absenteeism from work and school as well as marked loss in productivity with the most recent attack and in between attacks [159].

The Burden of Illness Study in Europe (Denmark, Germany, Spain) showed that HAE had a high impact on daily activities during attacks and also impacted patients’ daily activities between attacks [159–161]. Patients reported substantial anxiety about future attacks, traveling, and passing HAE to their children [161]. Attack severity was shown to be related to absenteeism [158, 159], and 51% (n = 84) of patients indicated that HAE had hindered their career/educational advancement [159].

European studies employing general HRQoL instruments also showed that attacks had a negative effect on HRQoL [162] including, specifically, attack frequency, as measured by the European Quality of Life 5 Dimensions Questionnaire (EQ-5D) [158] or the Short Form 36 Health Survey (SF-36) [163]. Prophylactic therapies, including lanadelumab and both IV and SC pdC1-INH, all demonstrated improvements in HRQoL [38, 114, 147, 152, 164].

An international specific HRQoL questionnaire for adult patients with HAE-1/2 has been developed called the HAE-QoL [165, 166]. During the development of the HAE-QoL, the factors cited most often by both experts and patients as affecting their QoL included potentially life-threatening attacks; the adverse side effects of medication (in several cases associated with chronic treatment); the unavailability of specific acute treatment at several healthcare centres; hereditary transmission; the lack of a known trigger which could be avoided; and the fact that it is a rare disease about which healthcare professionals know very little [165]. 
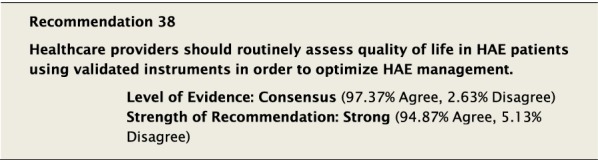


#### Clinical considerations

Assessment of HAE control as it relates to the frequency, duration and severity of attacks is not the only thing to consider when monitoring patients. Data suggests that factors which relate to a patient’s QoL are important when following patients with HAE. Although the HAE-QoL was developed specifically for adult HAE-1/2 patients, it has not been shown to be responsive when a 6-month recall period was used in one small study [167]. The Angioedema Quality of Life Questionnaire (AE-QoL) [168, 169] has been used in a large clinical trial, and although it was found to be responsive [152], it may not be specific enough to assess all the problems faced by patients with HAE. Validated instruments that are short, specific and responsive are needed to routinely assess HAE patients and optimize their management.

### Comprehensive care

#### Background

Comprehensive care of patients is based on integration of the organization, delivery, and management of services related to diagnosis, treatment, care, rehabilitation and health promotion. Many rare disease groups have adopted the comprehensive care model, and there is evidence in other rare diseases that this model results in better patient outcomes and reduced costs [143]. Haemophilia has used this model for decades. HAE is similar to other rare blood disorders, including haemophilia, because it is a chronic condition that is potentially life threatening and requires a highly specialized, multidisciplinary team to manage. However, although HAE is similar to other conditions, it is also different enough to require its own framework to meet the specific needs of these patients. The recommendation to provide comprehensive care for patients with HAE is not new and exists in previously published guidelines. The specific elements of comprehensive care for HAE were published previously and are listed in Table 4.
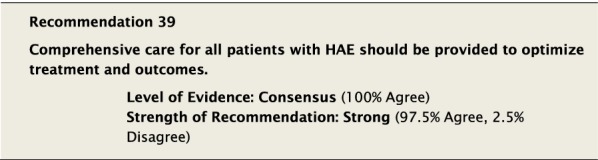

**Table 4:** Requirements for comprehensive care in the management of hereditary angioedema patients [9]

***Best Clinical Treatment outcomes including*** a. A comprehensive care team made up of nurse coordinator, clinician, social worker, data manager, pain management specialist, genetic counsellor, and administrative support b. Access to specialized diagnostic testing c. Access to home treatment d. A networked Patient Information System to facilitate product recalls—collect data on therapy outcome measures and safety, and facilitate participation in clinical trials e. Access to clinical advances as they become available f. Access to 24 hour support g. Access to up-to-date standards of care, including standardized wallet cards h. Tracking and intermittent audit of quality outcomes including beneficial and adverse outcomes through secure, comprehensive and networked data management ***Education of patients and staff regarding*** a. Responsible Self/Family Care (home care model) with home and self-infusion/administration instruction and support b. Developments in the cause, diagnosis, treatment, outcomes, and prognosis of HAE c. Changes in the administrative management of the clinic ***An environment conducive to research including*** a. Access to and support for clinical trials of new treatments b. Access to and support for translational research in diagnosis and prognosis c. Access to and support for psychosocial research such as quality of life studies	

#### Clinical considerations

Although the importance of the comprehensive care model in HAE was affirmed unanimously by Guideline Authors, and specific recommendations have existed with respect to its requirements, this care model is not available to all patients with HAE and every organization may adapt this model to its own context. Despite this, the fundamentals of comprehensive care should strive to be uniform and equally accessible within and between countries. Treatments for HAE can be expensive; however inappropriate treatment of HAE may be even more costly. Guideline Authors affirmed that ongoing monitoring of comprehensive care programs is essential to measure their efficacy as well as their impact on patients’ outcomes such as disease control, morbidity and mortality, hospital admissions, QoL, and economic effects. 
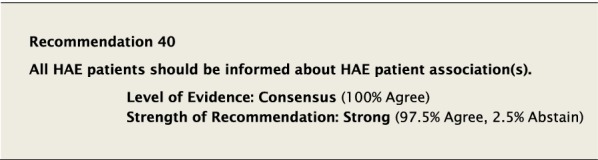


#### Clinical considerations

Patient organizations play a key role in supporting HAE patients and their caregivers. These organizations advocate for access to management and treatment for HAE patients, not only to control their disease, but to support them in fulfilling their potential at home, school, work, and in their relationships. All patients should be encouraged to join their local and international patient organizations.

### Registries

#### Background

Patient registries are a proven method for tracking clinical outcomes. Haemophilia, for example, has had vein-to-vein blood tracking since the early 1980s. Australian and Canadian bleeding disorder registries already exist for tracking the movement of blood products and provide a means for informing patients if any products they received have been recalled. As per the European Organisation for Rare Diseases (EURORDIS), the National Organization for Rare Disorders (NORD) and the Canadian Organization for Rare Disorders (CORD), rare disease patient registries should be recognized as a global priority in the field of rare diseases. Key principles of their joint declaration for rare disease registries include that registries should be centred on a disease group or group of diseases rather than a therapeutic intervention, they should encompass the widest geographic scope possible, and they should include data directly reported by patients as well as healthcare professionals [170]. A patient- and physician-driven global disease registry for HAE started in 2018 and is now recruiting [171].
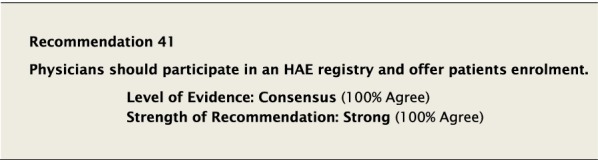


#### Clinical considerations

Patients enrolled in an HAE registry will have a traceable timeline of their treatment with blood products as well as a way to report adverse events through the registry. An HAE registry, especially a global one, will also provide a growing repository of data for research in the field with the aim to improve patient care.

## Conclusions

This update to the 2014 Canadian Hereditary Angioedema Guideline is the collaborative effort of Canadian and international hereditary angioedema (HAE) experts and patient groups led by the Canadian Hereditary Angioedema Network. It aims to optimize the management of patients with HAE worldwide by providing current, evidence-based recommendations to healthcare providers who are either managing patients with HAE or who are likely to encounter them in their practice.

## Supplementary information

**Additional file 1.** HAE RCT Evidence Tables. Randomized Controlled Trials. Includes data extraction, quality assessments, and study reference codes and citations for randomized controlled trials.

**Additional file 2.** HAE Lower Quality Comparison Study Evidence Tables. Lower Quality Comparison Studies. Includes data extraction, quality assessments, and study reference codes and citations for lower quality comparison studies.

## Data Availability

Data sharing is not applicable to this article as no datasets were generated or analysed during the current study.

## Appendices

### Appendix 1: Search strategy

Database: Ovid MEDLINE

Search conducted on June 27, 2018 and November 4, 2018 by Kelly Lang-Robertson, MLIS

Results previously identified in the October 2013 search conducted to support the 2014 guideline were manually removed from the new set of results.

#### Inclusion criteria

- English language publications
- Human subjects
- ≥ 5 subjects in each comparison group
- Original results published in an indexed journal (i.e., no data from abstracts/posters included)
  - Population: Patients diagnosed with type I or type II hereditary angioedema or hereditary angioedema with normal C1-INH of any age, including pregnant and pediatric populations
  - Intervention—addressed at least one of:
    - Acute treatment
      - C1-INH, rhC1-INH (ruconest), kallikrein inhibitors (ecallantide), bradykinin receptor antagonists (icatibant), antifibrinolytic drugs (tranexamic acid), solvent/detergent-treated plasma (SDP), fresh frozen plasma (FFP), frozen plasma (FP), epinephrine, corticosteroids, H1 antagonists, H2 antagonists
    - Long-term prophylaxis
      - C1-INH (IV or subcutaneous), attenuated androgens (danazol), antifibrinolytics (tranexamic acid), rhC1-INH (ruconest), synthetic steroids (tibolone), e-aminocaproic acid (EACA), oral kallikrein inhibitors (BCX7353, avoralstat), plasma kallikrein inhibitors (lanadelumab), anti-Factor XIIa antagonist monoclonal antibody (CSL-312)
    - Short-term prophylaxis
      - C1-INH (IV or subcutaneous), attenuated androgens (danazol), antifibrinolytics (tranexamic acid), anabolic steroids, fresh frozen plasma (FFP), frozen plasma (FP)
  - Comparison:
    - Any, including placebo, regular treatment, or no intervention
  - Outcome:
    - Frequency or severity of attacks, or symptom relief including at least one of:
      - Time to symptom relief (onset or complete resolution)
      - Mean symptom complex severity score (MSCS score)
      - Treatment outcome score (TOS)
      - Attack duration
      - Time to treatment
      - Rebound/relapse
      - Number of attacks (e.g., median per year or per month)
      - Health related quality of life
      - Work productivity activity index
      - Depression scores
      - Anxiety scores

Note: Intermediate outcomes such as laboratory measures are not included (e.g., mean serum concentrations of functional C1-INH).

### Appendix 2: Levels of evidence and strength of recommendation

Note: Levels of evidence (Table 5) and strength of recommendation (Table 6) were adapted from GRADE [20–22]. GRADE is considered ‘‘outcome centric,’’ and traditionally recommends a single rating for each outcome across the full body of evidence.

**Table 5:** Levels of evidence

Quality level	Definition
High	We are very confident that the true effect lies close to that of the estimate of the effect
Moderate	We are moderately confident in the effect estimate: The true effect is likely to be close to the estimate of the effect, but there is a possibility that it is substantially different
Low	Our confidence in the effect estimate is limited: The true effect may be substantially different from the estimate of the effect
Very Low	We have very little confidence in the effect estimate: The true effect is likely to be substantially different from the estimate of effect

If no published evidence was identified in an area, but Guideline Authors determined that it was important to make a recommendation, this was labeled as Consensus

**Table 6:** Strength of recommendation

Recommendations can be either STRONG or WEAK Strength of recommendation is determined by 1. Quality of evidence The higher the quality of evidence, the higher the likelihood that a Strong recommendation is warranted 2. Balance between desirable and undesirable effects The larger the difference between the desirable and undesirable effects, the higher the likelihood that a Strong recommendation is warranted 3. Values and Preferences The more values and preferences vary, or the greater the uncertainty in values and preferences, the higher the likelihood that a Weak recommendation is warranted 4. Costs (resource allocation) The higher the costs of an intervention—that is, the greater the resources consumed—the lower the likelihood that a Strong recommendation is warranted	

#### Determining levels of evidence

All non-randomized, non-blinded trials were considered to be Low level evidence.

All randomized controlled trials were considered to be High level evidence, and were downgraded based on the following parameters:

**Table:**

Parameters	Examples of limitations	Effect on level of evidence^a^
Limitations of design/risk of bias (Cochrane Risk of Bias tool)	Lack of allocation concealment Lack of adequate sequence generation Lack of blinding Incomplete data Selective reporting Other limitations such as stopping early	Majority of items not satisfied or not reported = downgraded by 1 level
Inconsistency	Large variation in effect Poor heterogeneity of results	Downgraded by 1 level
Imprecision of results	Small sample size Wide confidence intervals around the estimate of the effect Study is underpowered	Noted but not downgraded
Publication bias	Bias introduced due to significant industry funding	Noted but not downgraded (majority of studies were industry funded)
Indirectness/generalizability	Study population or setting differs significantly from population of interest	Not applicable

^a^If it was determined that the limitation was significant, the Level of Evidence was downgraded by 2 levels

#### Factors considered when deciding on a Strong or Weak recommendation

- Uncertainty in the estimates of likely benefit, and likely risk, inconvenience, and costs
- Importance of the outcome that treatment prevents
- Magnitude of treatment effect
- Precision of estimate of treatment effect
- Risks associated with therapy
- Burdens of therapy
- Risk of target event
- Costs
- Varying values

#### The implications of a Strong recommendation are

- Clinicians: Most patients should receive the recommended course of action
- Patients: Most people would want the recommended course of action and only a small proportion would not
- Policy makers: The recommendation can be adopted as a policy in most situations

#### The implications of a Weak recommendation are

- Clinicians: Different choices will be appropriate for different patients, and you must help each patient to arrive at a management decision consistent with her or his values and preferences
- Patients: Most would want the recommended course of action, but many would not
- Policy makers: Policy making will require substantial debate and the involvement of many stakeholders

## Abbreviations

ACEi: angiotensin-converting enzyme inhibitors
AE-QoL: Angioedema Quality of Life
AOHQ: Angio-Oedème Héréditaire du Québec
C1-INH: complement component 1 esterase inhibitor
C4: complement component 4
CHAEN: Canadian Hereditary Angioedema Network
COI: conflict of interest
CORD: Canadian Organization for Rare Disorders
EQ-5D: European Quality of Life 5 Dimensions
EURORDIS: European Organisation for Rare Diseases
FXII: coagulation factor 12
HADS: Hospital Anxiety and Depression Scale
HAE: hereditary angioedema
HAE-1: hereditary angioedema type 1
HAE-1/2: hereditary angioedema types 1 and 2
HAE-2: hereditary angioedema type 2
HAE-ANGPT1: hereditary angioedema angiopoietin 1 gene variant
HAE-FXII: hereditary angioedema coagulation factor 12 gene variant
HAE-PLG: hereditary angioedema plasminogen gene variant
HAE-UNK: hereditary angioedema unknown gene variant
HAEi: HAE International
HAE nC1-INH: hereditary angioedema with normal C1-inhibitor
HRQoL: health-related quality of life
ICMJE: International Committee of Medical Journal Editors
IgG1: immunoglobulin G1
IV: intravenous
LTP: long-term prophylaxis
NORD: National Organization for Rare Disorders
pdC1-INH: plasma-derived complement component 1 inhibitor
QoL: quality of life
RCAH: Réseau Canadien d’angioédème héréditaire
RCT: randomized controlled trial
rhC1-INH: recombinant human complement component 1 inhibitor
SC: subcutaneous
SDP: solvent detergent plasma
SF-36: Short Form 36 Health Survey
STP: short-term prophylaxis
U: international units
VAS: Visual Analog Scale
WHO: World Health Organization
