# Correction to: The International/Canadian Hereditary Angioedema Guideline

**DOI:** 10.1186/s13223-020-00430-4

**Published:** 2020-05-06

**Authors:** Stephen Betschel, Jacquie Badiou, Karen Binkley, Rozita Borici-Mazi, Jacques Hébert, Amin Kanani, Paul Keith, Gina Lacuesta, Susan Waserman, Bill Yang, Emel Aygören-Pürsün, Jonathan Bernstein, Konrad Bork, Teresa Caballero, Marco Cicardi, Timothy Craig, Henriette Farkas, Anete Grumach, Connie Katelaris, Hilary Longhurst, Marc Riedl, Bruce Zuraw, Magdelena Berger, Jean-Nicolas Boursiquot, Henrik Boysen, Anthony Castaldo, Hugo Chapdelaine, Lori Connors, Lisa Fu, Dawn Goodyear, Alison Haynes, Palinder Kamra, Harold Kim, Kelly Lang-Robertson, Eric Leith, Christine McCusker, Bill Moote, Andrew O’Keefe, Ibraheem Othman, Man-Chiu Poon, Bruce Ritchie, Charles St-Pierre, Donald Stark, Ellie Tsai

**Affiliations:** 1 University of Toronto, Toronto, ON Canada; 2 HAE Canada, Notre Dame Des Lourdes, MB Canada; 3 Department of Medicine, Queen’s University, Kingston, ON Canada; 4 Department of Medicine, Laval University, Quebec City, QC Canada; 5 Division of Allergy and Clinical Immunology, St. Paul’s Hospital, Department of Medicine, University of British Columbia, Vancouver, BC Canada; 6 Department of Medicine, McMaster University, Hamilton, ON Canada; 7 Department of Medicine, Dalhousie University, Halifax, NS Canada; 8 University of Ottawa Medical School, Ottawa, ON Canada; 9 Goethe-Universität Frankfurt am Main, Frankfurt Am Main, Germany; 10 Department of Internal Medicine, University of Cincinnati, Cincinnati, OH USA; 11 Department of Dermatology, University Hospital of the Johannes Gutenberg-University of Mainz, Mainz, Germany; 12 Hospital La Paz Institute for Health Research, Madrid, Spain; 13 Department of Internal Medicine, Universita degli Studi di Milano, Ospedale L. Sacco, Milan, Italy; 14 Departments of Medicine and Pediatrics, Penn State University, Hershey, PA USA; 15 3rd Department of Internal Medicine, Faculty of Medicine, Semmelweis University, Budapest, Hungary; 16 Laboratory of Clinical Immunology, Faculdade de Medicine ABC, Sao Paulo, Brazil; 17 Campbelltown Hospital, Western Sydney University, New South Wales, Australia; 18 ddenbrooke’s Hospital, Cambridge and University College Hospital, London, England, UK; 19 University of California, San Diego, San Diego, CA USA; 20 Moncton Hospital, Moncton, NB Canada; 21 Division of Allergy and Clinical Immunology, Centre Hospitalier Universitaire de Québec, Laval University, Quebec City, QC Canada; 22 HAE International (HAEi), Horsens, Denmark; 23 HAE International (HAEi), Fairfax, VA USA; 24 Institut de Recherches Cliniques de Montréal, Montreal, QC Canada; 25 Toronto Allergy Group, Toronto, ON Canada; 26 Southern Alberta Rare Blood and Bleeding Disorders Program, Foothills Medical Centre, University of Calgary, Calgary, AB Canada; 27 Division of Pediatrics, Faculty of Medicine, Memorial University, St John’s, NF Canada; 28 Janeway Children’s Health and Rehabilitation Centre, Memorial University, St John’s, NF Canada; 29 Division of Clinical Immunology and Allergy, Department of Medicine, Western University, London, ON Canada; 30 Division of Clinical Immunology and Allergy, Department of Medicine, McMaster University, Hamilton, ON Canada; 31 Department of Medicine, University of Toronto, Oakville, ON Canada; 32 Department of Immunology, McGill University Health Centre, Montreal, QC Canada; 33 Department of Medicine, Western University, London, ON Canada; 34 College of Medicine, University of Saskatchewan, Regina, SK Canada; 35 Departments of Medicine, Pediatrics and Oncology, University of Calgary Cumming School of Medicine, Calgary, AB Canada; 36 Departments of Medicine and Medical Oncology, University of Alberta, Edmonton, AB Canada; 37 L’angio-oedème Héréditaire du Québec, Quebec City, QC Canada; 38 Department of Medicine, University of British Columbia, Vancouver, BC Canada; 39 Department of Internal Medicine, Queen’s University, Kingston, ON Canada

## Correction to: Allergy Asthma Clin Immunol (2019) 15:72 10.1186/s13223-019-0376-8

Following the publication of this article [1], the authors requested to amend the characterisation of ‘lanadelumab’ from ‘humanised’ to the correct ‘fully human’.

Therefore, the fourth paragraph under Recommendation #24 should be amended to read:

“Lanadelumab (Takeda), a fully human monoclonal antibody against kallikrein, takes approximately 70 days to reach a steady state concentration [110], and is therefore not recommended for STP…”

Similarly, the first paragraph under Recommendation #27 should be amended to read:

“Lanadelumab is a subcutaneously injectable, fully human, anti-active plasma kallikrein monoclonal antibody (IgG1/κ-light chain)…”

## References

[1] Betschel S, Badiou J, Binkley K, Borici-Mazi R, Hébert J, Kanani A, Keith P, Lacuesta G, Waserman S, Yang B, Aygören-Pürsün E, Bernstein J, Bork K, Caballero T, Cicardi M, Craig T, Farkas H, Grumach A, Katelaris C, Longhurst H, Riedl M, Zuraw B, Berger M, Boursiquot J, Boysen H, Castaldo A, Chapdelaine H, Connors L, Lisa F, Goodyear D, Haynes A, Kamra P, Kim H, Lang-Robertson K, Leith E, McCusker C, Moote B, O’Keefe A, Othman I, Poon M, Ritchie B, St-Pierre C, Stark D, Tsai E (2019). The International/Canadian Hereditary Angioedema Guideline. Allergy Asthma Clin Immunol.

